# Supplementary material for: Isocitrate Dehydrogenase Alpha-1 Modulates Lifespan and Oxidative Stress Tolerance in Caenorhabditis elegans
Source: Int J Mol Sci. 2022 Dec 29;24(1):612. doi: 10.3390/ijms24010612 (PMC9820670; doi:10.3390/ijms24010612)
Supplement: Supplementary file 1 [file ijms-24-00612-s001.zip › ijms-2033854-supplementary.pdf]

Figure S1. The images of the GFP expression patterns driven under *idha-1* promoter in the *Pidha-1::gfp* transgenic worm by fluorescent microscopy.

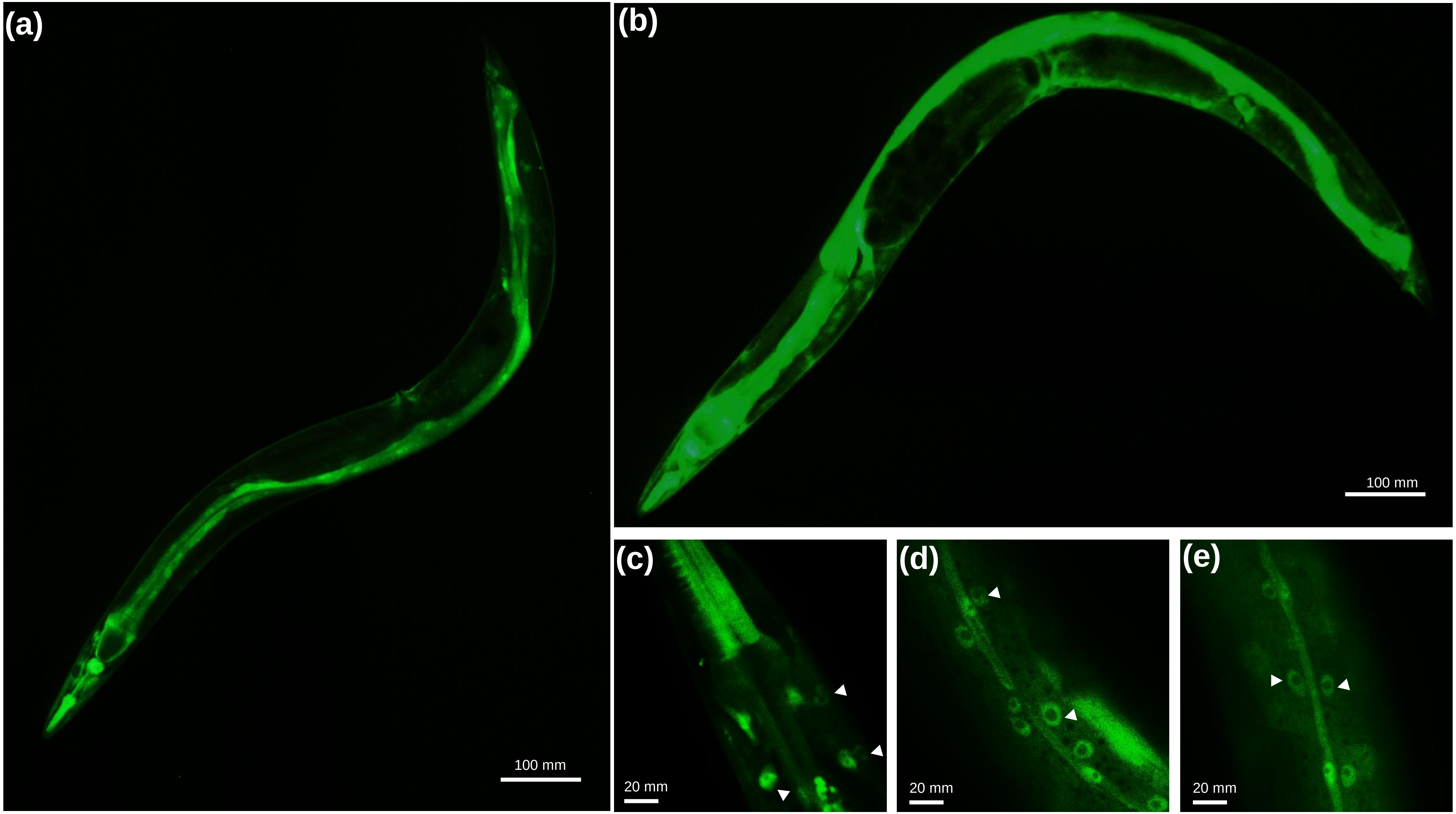

Figure S2. Upregulated *idha-1* mRNA levels are detected in the DR treatment and DR models.

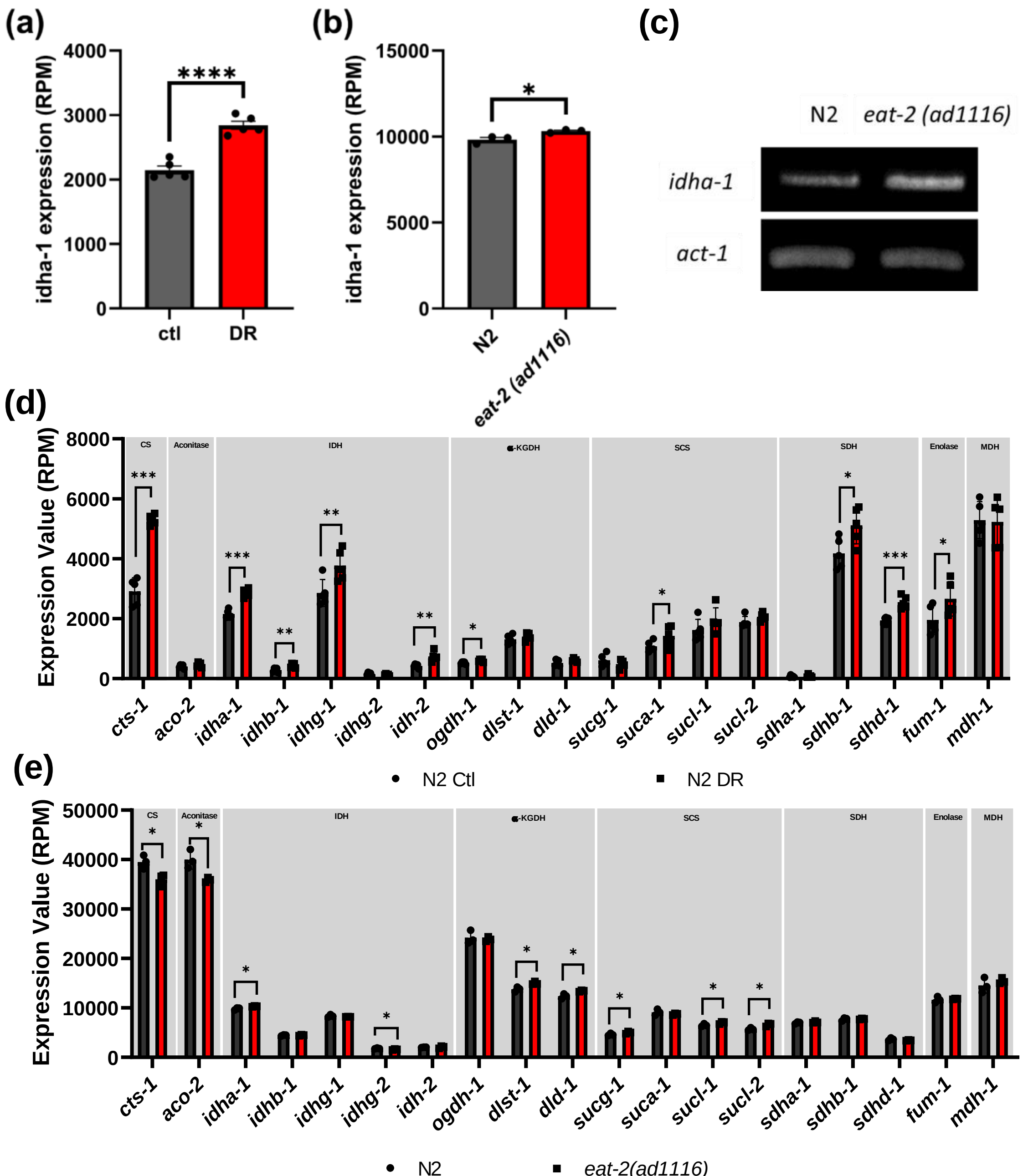

Figure S3. IDHA-1 is conserved among the *Caenorhabditis* genus.

(a)

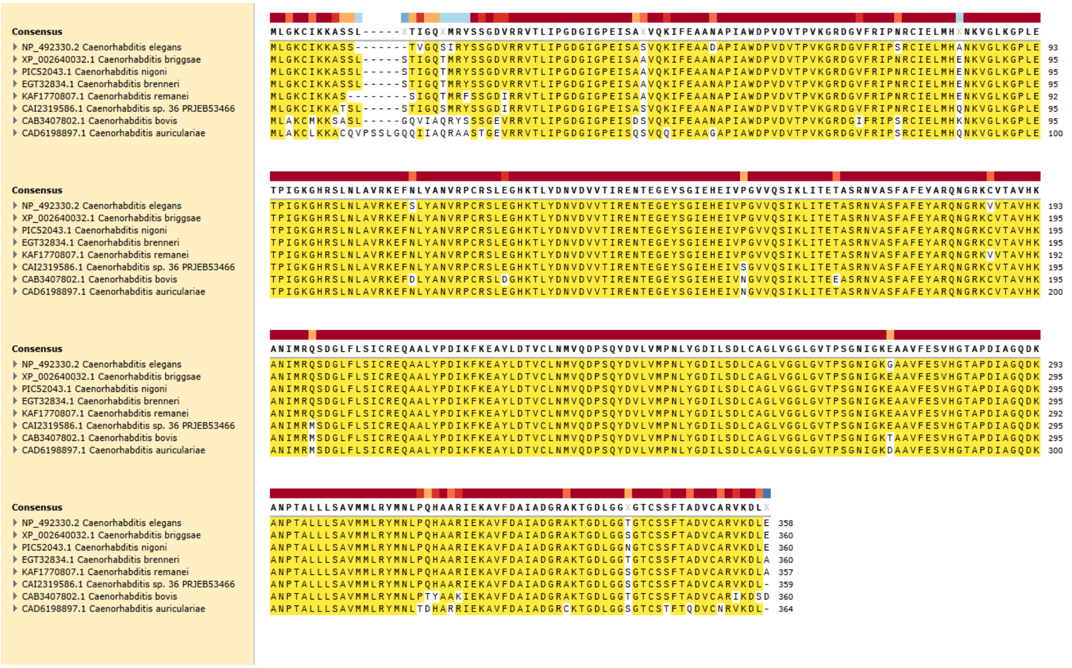

(b)

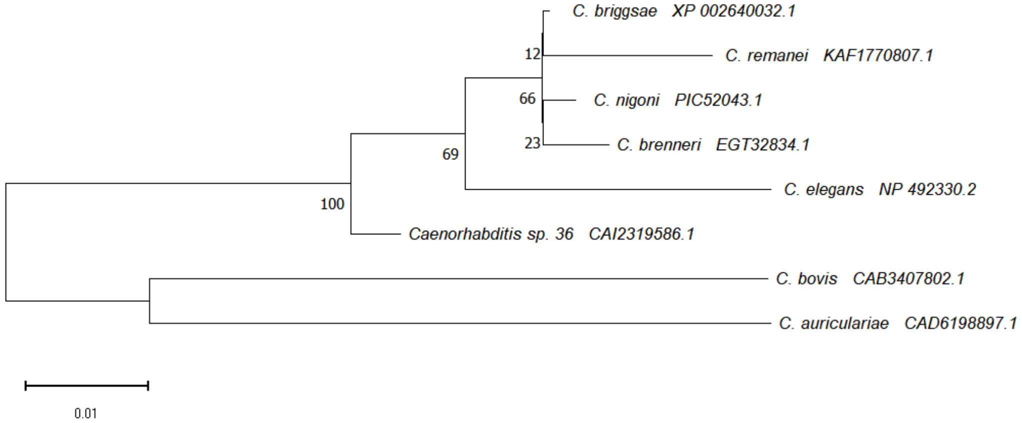

Figure S4. The elevated NAD+ levels are detected in *idha-1* overexpression transgenic worm.

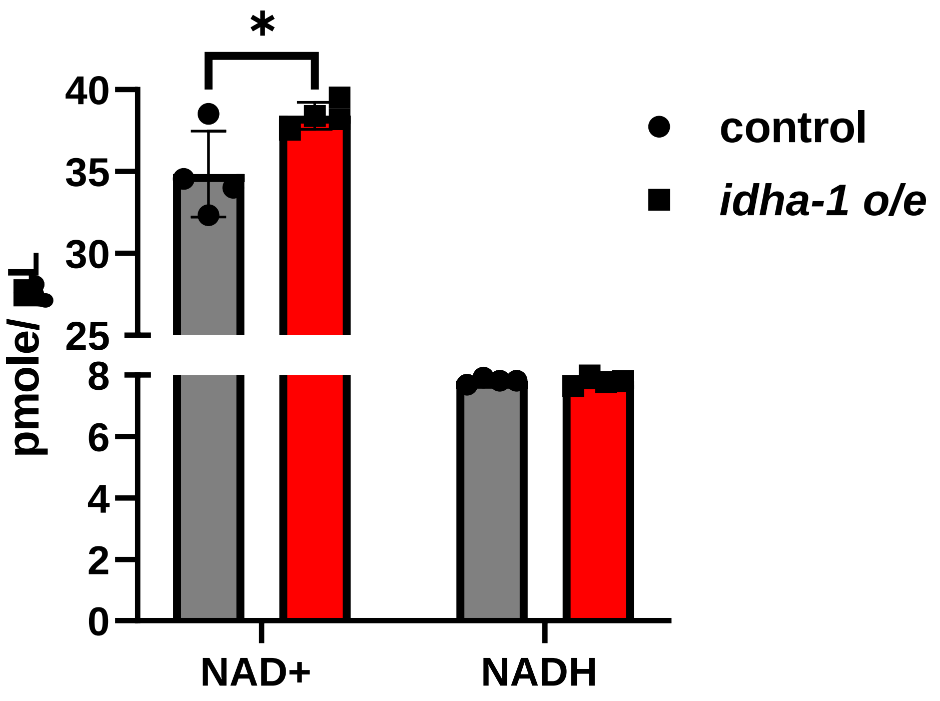

Table S1. The summary of the lifespan assays in figure 1a, 1e, 4c, 5a, 5b, 5c.

Strain	Mean lifespan (day)	Change (%)	p-value	Number of worms scored	Figure
Pidha-1::gfp	13.6	-	-	168	Fig. 1a
Pidha-1::idha-1	18.0	32.4	p<0.0001****	175	Fig. 1a
N2 *EV*	20.1	-	-	169	Fig. 1e
N2 *idha-1(RNAi) 5'*	16.8	-16.4	p<0.0001****	173	Fig. 1e
N2 *idha-1(RNAi) 3'*	16.3	-19.4	p<0.0001****	167	Fig. 1e
N2 *EV*	20.1	-	-	169	Fig. 4c
N2 *idha-1(RNAi) 5'*	16.8	-16.4	p<0.0001****	193	Fig. 4c
eat-2(ad1116) EV	25.7	-	-	113	Fig. 4c
eat-2(ad1116) idha-1(RNAi) 5'	20.8	-19.1	p<0.0001****	120	Fig. 4c
N2 *EV*	14.7	-	-	139	Fig. 5a
N2 *idha-1(RNAi) 5'*	11.3	-23.1	p<0.0001****	141	Fig. 5a
daf-16(mu86) EV	10.9	-25.9	p<0.0001****	121	Fig. 5a
daf-16(mu86) idha-1(RNAi) 5'	9.0	-38.8	p<0.0001****	120	Fig. 5a
N2 *EV*	17.2	-	-	139	Fig. 5b
N2 *idha-1(RNAi) 5'*	12.2	-29.1	p<0.0001****	141	Fig. 5b
aak-2(gt33) EV	12.2	-29.1	p<0.0001****	141	Fig. 5b
aak-2(gt33) idha-1(RNAi) 5'	10.8	-37.2	p<0.0001****	117	Fig. 5b
N2 *EV*	16.4	-	-	118	Fig. 5c
N2 *idha-1(RNAi) 5'*	12.1	-26.2	p<0.0001****	119	Fig. 5c
rsks-1(ok1255) EV	18.1	10.4	p<0.05*	122	Fig. 5c
rsks-1(ok1255) idha-1(RNAi) 5'	16.3	-0.6	p=0.052	122	Fig. 5c
